# 5-Iodo­pyrimidin-2-amine

**DOI:** 10.1107/S1600536810019124

**Published:** 2010-05-26

**Authors:** Yen-Hsun Chiang, Chia-Jun Wu, Pei-Chi Cheng, Jhy-Der Chen

**Affiliations:** a Department of Chemistry, Chung-Yuan Christian University, Chung-Li, Taiwan

## Abstract

The mol­ecule of the title compound, C_4_H_4_IN_3_, has crystallographic mirror plane symmetry. In the crystal, the mol­ecules are connected through N—H⋯N hydrogen bonds into polymeric tapes extended along the *a* axis, which are typical of 2-amino­pyrimidines. Each mol­ecule acts as a double donor and a double acceptor in the hydrogen bonding.

## Related literature

For coordination polymers formed with the title compound, see: Lin *et al.* (2006 ▶).
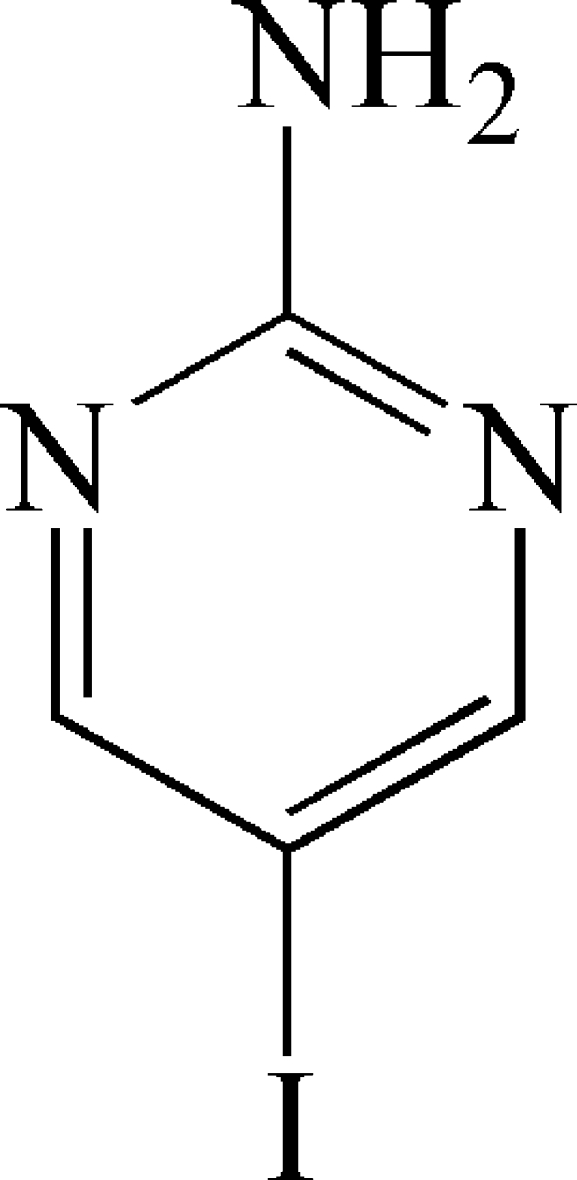


## Experimental

### 

#### Crystal data

C_4_H_4_IN_3_
*M* *_r_* = 221.00
Orthorhombic, 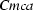

*a* = 7.9088 (7) Å
*b* = 8.3617 (10) Å
*c* = 18.3821 (16) Å
*V* = 1215.6 (2) Å^3^
*Z* = 8
Mo *K*α radiation
μ = 5.16 mm^−1^
*T* = 295 K
0.6 × 0.4 × 0.2 mm

#### Data collection

Bruker P4 diffractometer
Absorption correction: multi-scan (*XSCANS*; Siemens, 1995 ▶) *T*_min_ = 0.332, *T*_max_ = 1.000
800 measured reflections
573 independent reflections
535 reflections with *I* > 2σ(*I*)
*R*_int_ = 0.032
3 standard reflections every 97 reflections  intensity decay: none

#### Refinement

*R*[*F* ^2^ > 2σ(*F* ^2^)] = 0.032
*wR*(*F* ^2^) = 0.089
*S* = 1.10
573 reflections
48 parameters
H atoms treated by a mixture of independent and constrained refinement
Δρ_max_ = 0.93 e Å^−3^
Δρ_min_ = −0.83 e Å^−3^

### 

Data collection: *XSCANS* (Siemens, 1995 ▶); cell refinement: *XSCANS*; data reduction: *XSCANS*; program(s) used to solve structure: *SHELXS97* (Sheldrick, 2008 ▶); program(s) used to refine structure: *SHELXL97* (Sheldrick, 2008 ▶); molecular graphics: *XP* in *SHELXTL* (Sheldrick, 2008 ▶); software used to prepare material for publication: *SHELXTL*.

## Supplementary Material

Crystal structure: contains datablocks I, global. DOI: 10.1107/S1600536810019124/gk2275sup1.cif

Structure factors: contains datablocks I. DOI: 10.1107/S1600536810019124/gk2275Isup2.hkl

Additional supplementary materials:  crystallographic information; 3D view; checkCIF report

## Figures and Tables

**Table 1:** Hydrogen-bond geometry (Å, °)

*D*—H⋯*A*	*D*—H	H⋯*A*	*D*⋯*A*	*D*—H⋯*A*
N2—H2*N*⋯N1^i^	0.79 (5)	2.37 (5)	3.157 (4)	173 (6)

Symmetry code: (i) 
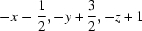
.

**Fig. 1.**
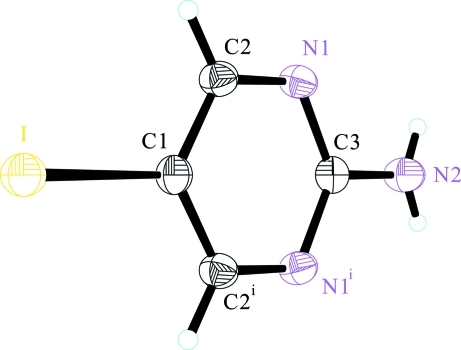
Crystal structure of the title compound with labeling and displacement ellipsoids drawn at the 30% probability level.Symmetry codes: (i) -x, y, z.

## supplementary crystallographic information

### Comment

A series of Ag(I) coordination polymers containg 2-amino-5-iodopyrimidine have been prepared, which show metallocycles and one-dimensional helical chains (Lin, *et al.*, 2006). Within this project the crystal structure of 2-amino-5-iodopyrimidine was determined to investigate its weak interactions.

In its crystal structure weak intermolecular N—H···N hydrogen bonding is found (Tab. 1) and the molecules are almost planar (Fig. 1).

### Experimental

The title compound was purchased from Acros Chemical Co. and used as received. Coloress plate crystals suitable for X-ray crystallography were obtained by dissolving the title compound in THF, followed by allowing the solution to evaporate slowly under air.

### Refinement

The pyrimidyl hydrogen atoms were placed into idealized positions and constrained by the riding atom approximation with *C*—H = 0.93 Å, and *U*_iso_(H) = 1.2 *U*_eq_(C). The amine hydrogen atoms were located from difference Fourier maps..

### Crystal data

**Table:**

C_4_H_4_IN_3_	*F*(000) = 816
*M**_r_* = 221.00	*D*_x_ = 2.415 Mg m^−^^3^
Orthorhombic, *C**m**c**a*	Mo *K*α radiation, λ = 0.71073 Å
Hall symbol: -C 2bc 2	Cell parameters from 31 reflections
*a* = 7.9088 (7) Å	θ = 4.9–12.6°
*b* = 8.3617 (10) Å	µ = 5.16 mm^−^^1^
*c* = 18.3821 (16) Å	*T* = 295 K
*V* = 1215.6 (2) Å^3^	Plate, colorless
*Z* = 8	0.6 × 0.4 × 0.2 mm

### Data collection

**Table:**

Bruker P4 diffractometer	535 reflections with *I* > 2σ(*I*)
Radiation source: fine-focus sealed tube	*R*_int_ = 0.032
graphite	θ_max_ = 25.0°, θ_min_ = 2.2°
ω scans	*h* = −1→9
Absorption correction: multi-scan (*XSCANS*; Siemens, 1995)	*k* = −1→9
*T*_min_ = 0.332, *T*_max_ = 1.000	*l* = −21→1
800 measured reflections	3 standard reflections every 97 reflections
573 independent reflections	intensity decay: none

### Refinement

**Table:**

Refinement on *F*^2^	Secondary atom site location: difference Fourier map
Least-squares matrix: full	Hydrogen site location: inferred from neighbouring sites
*R*[*F*^2^ > 2σ(*F*^2^)] = 0.032	H atoms treated by a mixture of independent and constrained refinement
*wR*(*F*^2^) = 0.089	*w* = 1/[σ^2^(*F*_o_^2^) + (0.055*P*)^2^ + 3.1925*P*] where *P* = (*F*_o_^2^ + 2*F*_c_^2^)/3
*S* = 1.10	(Δ/σ)_max_ = 0.001
573 reflections	Δρ_max_ = 0.93 e Å^−^^3^
48 parameters	Δρ_min_ = −0.83 e Å^−^^3^
0 restraints	Extinction correction: *SHELXL97* (Sheldrick, 2008), Fc^*^=kFc[1+0.001xFc^2^λ^3^/sin(2θ)]^-1/4^
Primary atom site location: structure-invariant direct methods	Extinction coefficient: 0.0148 (9)

### Special details

**Table:**

Experimental. Refinement of *F*^2^ against ALL reflections. The weighted *R*-factor *wR* and goodness of fit *S* are based on *F*^2^, conventional *R*-factors *R* are based on *F*, with *F* set to zero for negative *F*^2^. The threshold expression of *F*^2^ > σ(*F*^2^) is used only for calculating *R*-factors(gt) *etc*. and is not relevant to the choice of reflections for refinement. *R*-factors based on *F*^2^ are statistically about twice as large as those based on *F*, and *R*- factors based on ALL data will be even larger.
Geometry. All esds (except the esd in the dihedral angle between two l.s. planes) are estimated using the full covariance matrix. The cell esds are taken into account individually in the estimation of esds in distances, angles and torsion angles; correlations between esds in cell parameters are only used when they are defined by crystal symmetry. An approximate (isotropic) treatment of cell esds is used for estimating esds involving l.s. planes.
Refinement. Refinement of *F*^2^ against ALL reflections. The weighted *R*-factor wR and goodness of fit *S* are based on *F*^2^, conventional *R*-factors *R* are based on *F*, with *F* set to zero for negative *F*^2^. The threshold expression of *F*^2^ > σ(*F*^2^) is used only for calculating *R*-factors(gt) etc. and is not relevant to the choice of reflections for refinement. *R*-factors based on *F*^2^ are statistically about twice as large as those based on *F*, and *R*- factors based on ALL data will be even larger.

### Fractional atomic coordinates and isotropic or equivalent isotropic displacement parameters (Å^2^)

**Table:**

	*x*	*y*	*z*	*U*_iso_*/*U*_eq_	
I	0.0000	0.25315 (4)	0.72128 (2)	0.0462 (4)	
N1	−0.1515 (4)	0.6239 (4)	0.57261 (16)	0.0378 (8)	
N2	0.0000	0.8038 (8)	0.5044 (4)	0.0456 (13)	
C1	0.0000	0.4412 (6)	0.6466 (3)	0.0345 (11)	
C2	−0.1488 (5)	0.5037 (4)	0.6200 (2)	0.0367 (9)	
H2C	−0.2507	0.4604	0.6358	0.044*	
C3	0.0000	0.6791 (7)	0.5508 (3)	0.0343 (11)	
H2N	−0.085 (7)	0.831 (7)	0.485 (3)	0.061 (15)*	

### Atomic displacement parameters (Å^2^)

**Table:**

	*U*^11^	*U*^22^	*U*^33^	*U*^12^	*U*^13^	*U*^23^
I	0.0388 (4)	0.0504 (5)	0.0495 (5)	0.000	0.000	0.01869 (14)
N1	0.0321 (17)	0.0427 (17)	0.0385 (16)	0.0031 (14)	0.0005 (12)	0.0041 (13)
N2	0.039 (3)	0.053 (3)	0.045 (3)	0.000	0.000	0.017 (3)
C1	0.038 (3)	0.033 (2)	0.032 (2)	0.000	0.000	0.002 (2)
C2	0.0329 (19)	0.0406 (19)	0.036 (2)	−0.0011 (16)	0.0016 (15)	0.0034 (14)
C3	0.041 (3)	0.035 (3)	0.027 (2)	0.000	0.000	0.000 (2)

### Geometric parameters (Å, °)

**Table:**

I—C1	2.088 (5)	N2—H2N	0.79 (5)
N1—C2	1.331 (4)	C1—C2	1.377 (4)
N1—C3	1.346 (4)	C2—H2C	0.9300
N2—C3	1.346 (10)		
			
C2—N1—C3	116.1 (3)	N1—C2—H2C	118.9
C3—N2—H2N	120 (4)	C1—C2—H2C	118.9
C2^i^—C1—C2	117.4 (5)	N1^i^—C3—N1	125.9 (5)
C2—C1—I	121.3 (2)	N1^i^—C3—N2	117.0 (2)
N1—C2—C1	122.2 (4)		

Symmetry codes: (i) −*x*, *y*, *z*.

### Hydrogen-bond geometry (Å, °)

**Table:**

*D*—H···*A*	*D*—H	H···*A*	*D*···*A*	*D*—H···*A*
N2—H2N···N1^ii^	0.79 (5)	2.37 (5)	3.157 (4)	173 (6)

Symmetry codes: (ii) −*x*−1/2, −*y*+3/2, −*z*+1.
